# Policy Implementation Challenges and Barriers to Access Sexual and Reproductive Health Services Faced By People With Disabilities: An Intersectional Analysis of Policy Actors’ Perspectives in Post-Conflict Northern Uganda

**DOI:** 10.34172/ijhpm.2021.28

**Published:** 2021-04-13

**Authors:** Muriel Mac-Seing, Emmanuel Ochola, Martin Ogwang, Kate Zinszer, Christina Zarowsky

**Affiliations:** ^1^Social and Preventive Medicine Department, School of Public Health, Université de Montréal, Montreal, QC, Canada.; ^2^Centre de recherche en santé publique (CReSP), Université de Montréal et CIUSSS du Centre-Sudde-l’Île-de-Montréal, Montreal, QC, Canada.; ^3^Public Health Department, St-Mary’s Hospital, Lacor, Uganda.; ^4^Institutional Direction Department, St-Mary’s Hospital, Lacor, Uganda.; ^5^School of Public Health, University of Western Cape, Bellville, South Africa.

**Keywords:** Intersectionality-Based Policy Analysis, People With Disabilities, Sexual and Reproductive Health, Health Equity, Policy Implementation, Uganda

## Abstract

**Background:** Emerging from a 20-year armed conflict, Uganda adopted several laws and policies to protect the rights of people with disabilities, including their sexual and reproductive health (SRH) rights. However, the SRH rights of people with disabilities continue to be infringed in Uganda. We explored policy actors’ perceptions of existing pro-disability legislation and policy implementation, their perceptions of potential barriers experienced by people with disabilities in accessing and using SRH services in post-conflict Northern Uganda, and their recommendations on how to redress these inequities.

**Methods:** Through an intersectionality-informed approach, we conducted and thematically analysed 13 in-depth semi-structured interviews with macro level policy actors (national policy-makers and international and national organisations); seven focus groups (FGs) at meso level with 68 health service providers and representatives of disabled people’s organisations (DPOs); and a two-day participatory workshop on disability-sensitive health service provision for 34 healthcare providers.

**Results:** We identified four main themes: (1) legislation and policy implementation was fraught with numerous technical and financial challenges, coupled with lack of prioritisation of disability issues; (2) people with disabilities experienced multiple physical, attitudinal, communication, and structural barriers to access and use SRH services; (3) the conflict was perceived to have persisting impacts on the access to services; and (4) policy actors recommended concrete solutions to reduce health inequities faced by people with disabilities.

**Conclusion:** This study provides substantial evidence of the multilayered disadvantages people with disabilities face when using SRH services and the difficulty of implementing disability-focused policy in Uganda. Informed by an intersectionality approach, policy actors were able to identify concrete solutions and recommendations beyond the identification of problems. These recommendations can be acted upon in a practical road map to remove different types of barriers in the access to SRH services by people with disabilities, irrespective of their geographic location in Uganda.

## Background

**Key Messages**

** Implications for policy makers**

- An intersectionality-informed analysis goes beyond describing a problem. It enables policy actors and researchers to examine intersecting social identities, diverse sources of knowledge, and multilevel factors, and to consciously explore complex policy issues for transformative policy solutions.
- Pro-disability policy implementation challenges are multiple and people with disabilities still experience physical, attitudinal, communication, and structural barriers to access and use sexual and reproductive health (SRH) services in post-conflict Northern Uganda.
- Policy actors, including health service providers, disabled people’s organisations (DPOs), national and international organisations, and national policy-makers, proposed numerous recommendations and solutions which can be applied within the normative space created by the recent adoption of the 2019 Disability Act.
- The combination of these recommendations contributes to redress situations of social inequity and injustice, and advances Uganda’s progress towards the Sustainable Development Goals for universal health coverage.

** Implications for the public**

The fundamental rights of people with disabilities, including their sexual and reproductive health (SRH) rights, continue to be violated despite the existence of many laws and policies adopted to promote the rights of people with disabilities in Uganda. The study found that multiple forms of barriers and policy implementation challenges still exist, preventing people with disabilities from accessing and using SRH services. Many actionable solutions at individual, community and national levels exist and can be implemented to redress historic health inequities and injustice. People with and without disabilities, health service providers, civil society organisations (CSOs) and policy-makers have a renewed opportunity to contribute to concretely ‘leave no one behind’, as promoted by the Sustainable Development Goals.

More than 180 Member States have ratified the United Nations (UN) Convention on the Rights of Persons with Disabilities (CRPD), which aims to promote, protect and ensure the fundamental human rights of people with disabilities.^1^ The CRPD was adopted in 2006 and came into force in 2008 after two decades of negotiation among international organisations, activists, disabled people’s organisations (DPOs), and governments.^2^ According to the CRPD, people with disabilities are people “who have a long term physical, mental, intellectual or sensory impairments which in interaction with various barriers may hinder their full and effective participation in society on an equal basis with others.”^3^ Worldwide, one person in seven is estimated to live with some form of disability, with 80% of them living in low and middle resource income countries.^4^ In 2019, the UN report on the realisation of the Sustainable Development Goals stated that despite improvement in development, people with disabilities continue to experience exclusion and face numerous barriers to their full participation.^5^

 In sub-Saharan Africa, Uganda cited as an exemplary disability rights promoter,^6,7^ was among the first countries to ratify the CRDP in 2008.^1^ One fifth of its population was estimated to live with some disability.^8^ In 1995, Uganda enacted its Constitution, and in 2005 it was amended, providing a legal space for the promotion of people with disabilities’ rights. In the following years, several legal instruments that contain sections or articles related to the rights of people with disabilities were adopted. Among these, Uganda approved the Parliamentary Election Statute in 1996 and the Local Government Act in 1997. These laws, respectively, make provision for people with disabilities to be elected to Parliament, and at the district and subcounty levels.^6^ In 2003, the National Council for Disability (NCD) Act was adopted and specified the role of this national body in the promotion, monitoring, and advocacy of equal opportunities for Ugandans with disabilities.^9^ Three years later, Uganda further adopted a Disability Act with sections related to such as accessibility, social services, and health, including access to reproductive health and user-friendly health facility materials.^10^ In September 2019, Uganda updated this Act with a more comprehensive version, referencing the CRPD and using a similar definition of disability.^11^

 Emerging from a 20-year armed conflict which most affected its Northern region, Uganda had to rebuild a weakened health system. It witnessed high levels of sexual and gender-based violence and unwanted pregnancies as well as poor access to safe motherhood^12,13^ and reproductive healthcare.^14^ Despite an arsenal of well-intentioned legal tools adopted over several years to promote and protect the human rights of people with disabilities, including their sexual and reproductive health (SRH) rights, people with disabilities continue to have limited access to routinely accessible SRH services in Uganda. Studies examining SRH service utilisation reported ongoing physical and costs barriers,^15,16^ attitudinal challenges,^15^ and multilayered discrimination and inequities^17^ experienced by people with disabilities. The 2018 Guttmacher-Lancet Commission also highlighted that people with disabilities constitute a group ‘with specific disadvantages’ and are ‘subjected to harmful stereotypes and myths’ which contribute to their heightened risk of physical and sexual abuse.^18^

 Intersectionality-Based Policy Analysis (IBPA) can critically address social inequities and multiple discriminations experienced by people with disabilities.^19^ It provides a flexible framework to enable policy actors, researchers, and group advocates to examine diverse sources of knowledge, intersecting multiple social identities and multilevel factors, and to explore complex policy issues for transformative policy solutions, beyond describing the problem.^20^ Intersectionality addresses the interrelationships among multiple social identities, social inequities, power dynamics, context, and complexity.^21^ Principles promoted in the IBPA are the importance of acknowledging intersecting social categories, a multilevel analysis, power structures, the context, the diversity of sources of knowledge, reflexivity, and social justice and equity.^22^ Before critical studies started to be interested in intersectionality to highlight inequality and multiple oppressions experienced by marginalised groups,^23-27^ Black feminists and lesbians of the Combahee River Collective were already embracing core concepts of this framework and approach in their struggle in the 1970s.^28^ Intersectionality was first coined in 1989 by Kimberlé Crenshaw to address the multiple discriminations faced by African American women workers who were protected by neither anti-racism nor anti-sexism legislation.^24,29^

 The study reported here aimed to understand and document how policy actors perceive the relationships among legislation and health policy and the utilisation of SRH services by people with disabilities in the post-conflict Northern region of Uganda. We were interested in exploring policy actors’ understanding of existing pro-disability legislation and policy implementation, their perceptions of possible discriminations experienced by people with disabilities in accessing and using SRH services, and their recommendations on how to redress these inequities. This paper reports the qualitative findings on the perceptions of policy actors at meso and macro levels, drawing from a larger body of evidence from a mixed methods study which also involved women and men with disabilities (micro level). Perspectives of women and men with disabilities have been reported previously.^17^

## Methods

 The qualitative study methods are reported in detail elsewhere and summarised here.^17^ From November 2017 to April 2018, we conducted our study in the districts of Gulu, Amuru, and Omoro in the Northern region and in Kampala, the capital of Uganda. To assess the rigour of our qualitative research, we followed the Consolidated Criteria for Reporting Qualitative Research.^30^

###  Study Participants

 A total of 115 people participated in the study: at the national level, 13 policy actors took part in in-depth semi-structured interviews; and at the community level, 68 health service providers and DPO representatives participated in seven focus groups (FGs) of the Northern districts of Gulu, Amuru, and Omoro. Additionally, 34 health service providers and managers participated in a 2-day participatory workshop on disability-sensitive health service provision (Table 1). Participants were purposefully recruited, following a snow-ball approach in which initially recruited study respondents recommended other potentially relevant policy actors that could speak to the research objectives.^31^ National policy actors based in the capital of Kampala were selected based on different types of organisations they belonged to and who were knowledgeable of disability and SRH related policy and programmatic processes in Uganda. Health service providers were recruited from seven health facilities, with a balance of gender and public and private-not-for-profit health facilities. Recruitment of study participants continued until saturation was reached.^32^

**Table 1:** Sample Characteristics

**Source**	**Total**	**Women (%)**	**Men(%)**	**Disabled (%)**
National level policy actors	13	6 (46)	7 (54)	5 (39)
Policy-makers from government	6	3	3	3
Representatives of international organisations/NGOs and national NGOs	7	5	2	2
Community level policy actors	68	36 (53)	32 (47)	7 (9)
Health service providers	60	34	26	1
Representatives of DPOs	8	2	6	6
Workshop participants	34	19 (56)	15 (44)	0 (0)
Health staff	27	16	11	0
Health managers	7	3	4	0
Total	115	61 (53)	54 (47)	12 (10)

Abbreviations: DPOs, disabled people’s organisations; NGOs, non-governmental organisations. *Note*: Due to rounding of percentage, the total might slightly be over or below 100.

###  Data Collection 

 We used in-depth semi-structured interviews, FGs, and a participatory workshop to triangulate findings.^32^ These techniques were selected to further increase the trustworthiness of qualitative research process.^32^ For fine-tuning of data collection tools, we first discussed them among the research team, and pre-tested each tool in FGs and with sign language interpreters to improve comprehension. The IBPA framework informed this research and was adapted in our interview and FG guidelines, which included two sets of questions^20^: The first set constituted of descriptive questions related to the identification of problems related to SRH use among people with disabilities and information on policy implementation processes. The second set was composed of transformative questions related to solutions aimed at reducing inequities and addressing problems identified. Individual and group interviews were conducted in English, and research assistants translated concurrently questions and answers in Luo, when needed. For the few participants with hearing impairments, we hired locally qualified Ugandan sign language interpreters who were fluent in English, Luo and sign language. Each individual or group interview lasted around 60 minutes and was audio recorded with the permission of study participants.

 Consistent with the transformative component of the IBPA emphasising the search for solutions, we organised a 2-day participatory workshop on disability-sensitive service provision, following the numerous requests we received from interviewed health service providers. The workshop objective was to discuss the barriers people with disabilities encountered when seeking SRH services and the solutions to address concretely these problems. It was organised for health service providers and managers of seven health facilities of three districts. On the first day, the preliminary findings of the study and the existing pro-disability policies and legislation in Uganda were presented. Two women, one with a physical impairment and another with a mental impairment, and two men, one with a hearing impairment and the second with a vision impairment, were invited as experts to share their experiences and recommendations on how to improve accessibility and service delivery. On the second day, a deaf trainer and a hearing trainer who knew sign language facilitated a series of hands-on sessions for participants to learn the basics of Uganda sign language in relation to health and SRH services. With the permission of workshop participants, we documented the outcomes of group discussions and exchanges.

 To ensure confidentiality, all citations from study respondents have been depersonalised and are referred to in this paper by their professional function only. For health service providers, the FG number is specified.

###  Analysis 

 Informed by the intersectional framework, a thematic analysis was adopted due to its flexible approach as well as the opportunity this type of analysis provides us in managing “a large data set” in a more structured manner.^33^ Our thematic analysis following specific steps which are described in detail elsewhere,^17^ and briefly summarised here. Relevant themes emerged after a series of iterative activities which included listening to all recordings, reading a couple of times all interview transcripts, and writing down analytical memos along the process. Transcripts were coded through QDA Miner 5.0.31 (Provalis) following an inductive and deductive approach. Our analysis was guided by the key principles of the IBPA framework of intersecting social categories, multilevel analysis (Figure), power structures, time and space (context), diverse sources of knowledge, reflexivity, and equity and social justice.^22^ Specifically, when identifying themes, we were attentive to how policy actors answered descriptive and transformative questions posed during individual and group interviews as well as during the workshop.^22^

**Figure:**
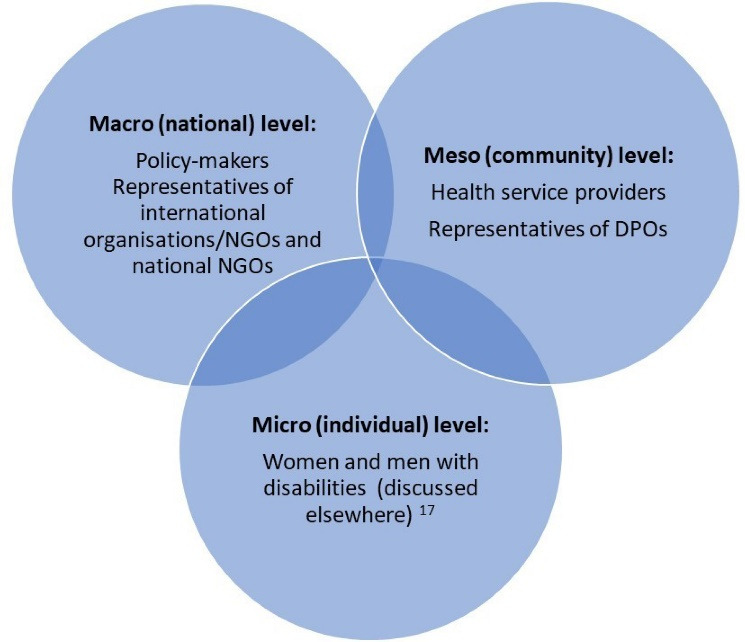
Multilevel Analysis of Policy Actors. Abbreviations: DPOs, disabled people’s organisations; NGOs, non-governmental organisations.

## Results

 We report here the findings of the perceptions of health service providers and representatives of DPOs, at meso level, and those of policy-makers and representatives of international organisations/non-governmental organisations (NGOs) and national NGOs, at macro level. They complement the findings of a larger body of evidence on the perceptions and recommendations of women and men with different types of impairments, at micro level, which highlighted the intersectional discriminations experienced by people with disabilities when using different types of SRH services.^17^ Although we interviewed diverse policy actors at meso and macro levels, they shared several common narratives around the relationships between pro-disability legislation and policy and the use of SRH services by people with disabilities in the post-conflict Northern region of Uganda.

 This study identified four major themes across policy actors, levels, and districts, as follows: (1) policy and legislation application challenges; (2) acknowledgment of the existence of multiple barriers faced by people with disabilities in accessing and using SRH services; (3) lingering impacts of the conflict on people with disabilities’ access to services; and (4) multilevel recommendations to remove barriers.

###  Policy and Legislation Application Challenges 

 Policy actors mentioned several challenges related to the implementation and enforcement of pro-disability policy and legislation in Uganda. Central to a lack of enforcement is a widespread lack of awareness and training on disability issues among policy executors, particularly health professionals, of existing key policy and laws which focus on the rights of people with disabilities. To some health service providers, this implementation gap was illustrated by inaccessible services and infrastructures.

*“It’s unfortunate that most of these things [policies] stop at Kampala or in offices. They [policy-makers] don’t come to the ground…. Myself, I have even never seen the [Disability] Act … This is something that they should also consider if it must work out very well … because we should work with references … It was not availed ….We also try to improvise. It is there, though it’s not [up] to standard. But it is a requirement that we should at least create accessible [structures] … It’s not very functional because some of our clients … are still crawling” *(Health service provider, FG9, Omoro).

 Awareness was identified as essential in the pathway to policy implementation; however, a lack of prioritisation and budgeting were also identified as detrimental to an effective response to disability issues from ministry to local levels. The deficient financial capacity at governmental level was perceived to be influenced by policy-makers’ worldviews and their lack of sensitivity towards disability issues.

*“I mean the issue of mindset has affected most of our implementation. If I showed you the percentage for the [disability] budget … Like the law (…) it should be backed by resources, financially. If it is a government building, let’s make sure it’s accessible. That means you need money to change … Of the Ministry, I think it’s 0.1, is it 1% or something less than 1%?”* (Government policy-maker, Kampala).

 Although Uganda has adopted many policies promoting the rights of people with disabilities, policy actors insisted on the importance of supervision and monitoring: “*There is no committee in place to supervise the policies that have been approved, so it is upon the organisation to take it on or not*” (Health service provider, FG4, Gulu). According to policy actors, the 2006 Disability Act was not substantial enough to hold the Government imputable to its policy intent: “*People have raised the issue that the Act has so many things missing … [The Act] doesn’t hold the Government accountable*” (Government policy-maker, Kampala). They further mentioned that the NCD and the civil society organisations (CSOs) were not fully playing their role of advocacy for and monitoring of accessible services for people with disabilities: “*It’s the role of disability unions and umbrellas to ensure that they engage institutions so that they can sign some memorandum of understanding … help push for disability-friendly services*” (National NGO representative, Kampala).

###  Acknowledgment of the Existence of Multiple Barriers Faced by People With Disabilities in Accessing and Using SRH Services

 Irrespective of their background and function, policy actors at both community and national levels reported similar barriers regarding the access to and utilisation of SRH services experienced by people with disabilities. Four types of barriers were identified: physical, attitudinal, communication, and structural (related to systems, policies, and norms). According to respondents, the lack of accessible equipment and infrastructure, such as toilets, was prevalent and prevented people with disabilities, especially women, from having optimal access to maternal and reproductive health services. Health service providers were frank about the physical accessibility gaps that they observed in their health facility, prompting some of them to revisit their service delivery approach.

*“Especially in our … maternity ward. You find that it is very hard to deliver them. Sometimes, we prefer to deliver them down on the floor. Sometimes, if you have the energy, you, as the medical person, you have to lift her up on the bed. She delivers. Again, you lift her down or you use a trolley to push her … In case of an operation ….We don’t have the equipment for people with [physical] disabilities like [involving] lower limbs. There is no way you can help her… [For] most of them, we deliver them on the floor. The delivery bed is made for normal people ….That is one of the challenges we’re having” *(Health service provider, FG4, Gulu).

 At the attitudinal level, participants reported that for many health service providers in Uganda, people with disabilities were perceived to be sexually inactive and incapable of entertaining sexual activities or having children. This common perception lead health staff to believe that people with disabilities did not need to use any SRH services. This ableist attitude could deny people with disabilities the possibility of receiving SRH services like anyone else.

*“[The] majority of them [health service providers] do not think disabled people are clients for reproductive health services … They imagine they might not need these services … I just asked them a question and I said: ‘If I come here with a wheelchair, rolling into your health centre, what will you see?, ’ they told me: ‘We see a wheelchair!’ ‘So, you don’t see the person?!’ … I said ‘It’s just your work to check whether the baby is lying there [she was pregnant at that time], and not to look at my disability. It’s the leg that is disabled … My womb is okay!’” *(Government policy-maker with a disability, Kampala).

 According to representatives of DPOs, most of whom were also people living with different impairments, the negative and discriminatory attitudes of health service providers were of concern. Often, these attitudes acted as deterrents among people with disabilities to seek care for health conditions that would necessitate medical attention. Moreover, DPO representatives questioned the professional ethics of health staff when they were providing SRH services.

*“I don’t know whether that is part of their code of conduct, but most of them are arrogant to clients at the hospital. This is a big barrier because most of our persons with disabilities would not want to go to [the] hospital where they are shouted at. In most cases, our health service providers do not know how to take care of [people with disabilities]. I think [that is] a very big barrier in accessing sexual health [and] reproductive services” *(DPO representative, Gulu).

 When further probed, most policy actors mentioned the communication barriers which people with hearing impairments faced. In Uganda, sign language is officially recognised. According to the Disability Act, sign language should be “introduced into the curriculum of medical personnel.”^10^ Interviewed health service providers reported receiving no training in this regard during their professional training or continued education opportunities. The inability of health service providers to communicate health information or instructions to people with hearing impairments led to sub-optimal provision of SRH care. These situations could be detrimental to people with disabilities and frustrating to health service providers who needed to find alternatives to understand the needs of people with hearing impairments.

*“Last week, we received one [patient who was a] deaf person. The problem was how to help? Because they use sign language … but none of us has been trained … I was trying to handle, doing signs … but I know [figured out] what she wanted because she came with a paper for HIV test … But when I wanted to talk to her, she cannot understand … I took her to the [HIV] counsellor, [but] I don’t know how they handled it” *(Health service provider, FG6, Omoro).

 At a structural level, one of the most important barriers policy actors reported was the lack of disability data collection and monitoring of service delivery. Although the Ministry of Health included a specific column on disability (Yes/No) in the patient registry book made available throughout health facilities in the country, this information was seldom or inconsistently collected. Most of health service providers did not receive any training on how to obtain and use data on disability nor did analyse the information collected when this was done.

*“We realised that we are not capturing our data well. And if [it] is captured … we are not reporting … As you report something, you should be able to analyse, and you put in practice … At least, there should be a strategy where … even in the district level, [and at] the facility [level], we should be able to generate the number of people who are having disability, so … it can help with planning. We don’t know how many clients we have who are disabled” *(Health service provider, FG9, Omoro).

 During the workshop, participants had the opportunity to learn directly from people with disabilities who acted as experts in their SRH care trajectory. The four people with disabilities explained who they were in their community, what happened to them when seeking health services, and how they were often mistreated by health service providers. They also described the multiple barriers they faced. While sharing their stories, they also made sure that health staff recognised their strengths and resilience, beyond their impairments and the limitations they were facing due to systemic obstacles, at environmental, attitudinal, and communication levels. According to workshop participants, this workshop helped them to be more reflexive and enabled them to better understand the situations of people with disabilities.

*“I want to apologise. We have been working on people with different disabilities, but we didn’t know what you people were going through. I want you to forgive me and us, the health workers … I would like to tell you that we shall see that we change the quality of care because disability can come to anyone, any time. So, I want you not to think that you are different from us. We shall make a change, I promise”* (Workshop participant, health manager, Gulu).

###  Lingering Impacts of the Conflict on People With Disabilities’ Access to Services

 For many respondents, the impacts of the conflict were still vivid despite the end of the conflict through a signed agreement between the Government and the Lord’s Resistance Army rebels in 2006.^34^ According to them, the armed conflict contributed to “the breakdown of the formal system” (International NGO representative, Kampala), and generated widespread disabilities and trauma for Northern Ugandans. It affected family structure, with persisting sequelae to date. Others mentioned the high level of gender-based violence which occurred during the conflict. Many young women and girls became “*child mothers*,” after being raped “*in the bush*,” a term referencing the period in rebel captivity (Health service provider, FG3, Amuru). These situations were compounded by limited access to SRH services: “*Access to all health services or reproductive health services for people with disabilities is [was] not easily accessible. And it’s worst in Northern Uganda. This is [was] due to war*” (National NGO representative, Kampala).

 Policy actors believed that the impacts of the conflict were “*worse for people with disabilities*” (International NGO representative, Kampala), especially “*women with disabilities in the North [who] are still recovering from war*” (Government policy-maker, Kampala). In the context of insecurity due to armed conflict, families, in some instances, had to save their own lives amid the fighting, leaving their relatives with disabilities behind: “*So, if you’re disabled and you have all these sorts of needs, and the family has to decide between running away to safety and helping you to access a service?*” (National NGO representative, Kampala). Paradoxically, while the conflict created different forms of hardship for people with and without disabilities, camps that were erected to cater for internally displaced Northern Ugandan populations also became a source of support considered as *“[one stop] shop centre[s]*” (Health service provider, FG9, Omoro) where all services such as food, education, and healthcare were provided for free, for all. However, as the conflict ceased, many NGOs stopped providing their humanitarian services, and people, including many with disabilities, had to fend for themselves and survive without any support.

*“But after that [the conflict], people were dispersed… They are now coming from different places. [This situation has] created distance from points of service delivery. If a crippled person has to move for more than 10 km to seek for healthcare, that has become very hard. I would say that this is negative to them because it is not very easy for them now to access services, as it used to [be in the camps]”* (Health service provider, FG9, Omoro).

 Based on the accounts of a few policy actors, a life spent in camps not only provided immediate benefits such as accessible and free services but also generated long-term social negative consequences. According to them, people lost their social compass and became dependent upon external sources to receive services. This situation might have created other social consequences given the lack of accessible services of proximity, including healthcare.

*“The post-war effect in Northern Uganda has been there. [There] is still [a] dependency syndrome. We had so many NGOs which were supporting the household activities. Most NGOs have gone away, so people have [feel] the effects now. People resorted to drinking…. We have child-headed families because of … loss of parents … loss of dear ones” *(Heath service provider, FG3, Amuru).

###  Multilevel Recommendations to Remove Barriers 

 At both community and national levels, policy actors described in detail the multiple barriers people with disabilities encountered when using health and SRH services. On the other hand, policy actors also identified specific recommendations to redress these barriers and better promote the rights of people with disabilities as enshrined in adopted policies and laws. Policy actors were reflective about their shortcomings, but they also went beyond listing problems. They felt the urgency to instill measures in their institution and capitalise on the strengths of people with disabilities to induce change.

*“My recommendation goes to the Quality Assurance team [of the hospital] … Concerning people with disabilities, much has not yet been done. So, I would advise that we get a committee that looks at the welfare of persons with disabilities, to see that this kind of training should be continuous. And disabled who are doing good things like these ones [people with disabilities invited in the workshop as experts] should be used as role models to the other disabled persons” *(Workshop participant, health service provider, Gulu).

 Given the intersectoral and multilayered nature of barriers to access SRH services and policy and legislation implementation challenges identified, policy actors acknowledged that solutions did not lie at a single location, nor could they be addressed by only one actor. Rather, respondents recommended solutions targeting specific policy actors. At the micro level, people with disabilities and their families were named, highlighting the importance of empowerment and the exercise of the basic rights of people with disabilities. At the meso level, both health service providers and local CSOs were mentioned as playing a crucial role in concretely removing barriers and in defending the rights of people with disabilities they served. Respondents argued that at the macro level, the Government and elected bodies held a prime position of being held accountable and responsible for putting in place actionable measures such as devoting financial and technical resources to mainstream disability in service delivery, including SRH services. The NCD was pinpointed as pivotal in monitoring the Government’s policy and legislation focusing on the promotion and protection of disability rights. National and international CSOs identified the need for more research and disability data collection and analysis for improved planning of services for people with disabilities.

Table 2 summarises the main recommendations made by policy actors at community and national levels during the interviews, FGs, and participatory workshop, targeting the three levels of actors at micro, meso, and macro levels.

**Table 2:** Main Areas of Recommendations Proposed for and by Policy Actors to Improve the Access to and Utilisation of SRH Services by People With Disabilities

**Levels**	**Recommendations **
**At micro level** People with disabilities and families	Awareness-raising of people with disabilities and their families on disability rights: *“Awareness-raising is needed at all levels from family, health staff to policy-makers”* (Government policy-maker, Kampala). Empowerment of people with disabilities and development of their leadership: *“Using people with disabilities themselves, train them. Use them to target their membership, that would be key”* (DPO representative, Gulu).
**At meso level** Health service providers and local authorities	Improvement of accessibility for disability-sensitive health-related infrastructure, equipment, and services: *“The delivery beds... When I went to the Midwifery Day in Fort Portal, Karamoja district, they came with a bed that I have never seen… those are the beds [for] the use for people with disabilities… They [policy-makers] could come to the hospital, [and] find out if there are people who are interested in learning sign language… We wait for these things to be integrated into our curriculums” *(Health service provider, FG2, Omoro).
Local CSOs	Advocacy for and representation of people with disabilities’ rights: *“… to create a network of people working in the area of disability so that we can have a unified voice to address the issues, not only health issues but other social issues that affect people with disabilities”* (DPO representative, Gulu).
**At macro level** Government	Monitoring and evaluation of policy processes: *“The Government needs to [have a] committee in action on the implementation of the existing laws. The Government must ensure that the accessibility must be universal… to all… They should widen their scope of consultations when they are coming up with their policies and guidelines… so that you can be in position to intersect, and also ensure that the needs of all the categories of people, whom you have consulted, are taken care of” *(DPO representative, Kampala). Disability mainstreaming with specific budget allocation: *“One priority is to mainstream disability at all levels of MCH [maternal and child health] and SRH, in all levels, but don’t separate people with disabilities. It should be integrated, data collected, and with a budget!” *(International organisation representative, Kampala).
NCD	Structural strengthening of the NCD: “*[There are] too many small disability organisations, and poorly coordinated. The NCD is not strong because too small…. Competition disadvantages, this decreases their bargaining power… If they are together at the same time, they have more power to ask for change. So, they need to be strong and give a united voice from all categories of people with disabilities for advocacy and lobbying” *(Government policy-maker, Kampala).
National and international CSOs	More systematic research and data collection on disability issues: *“The role of research… is to make sure you collect the appropriate and relevant data [which can] inform the service institutions so that they create the demand of services for [people with] disabilities”* (National NGO representative, Kampala).

Abbreviations: DPO, disabled people’s organisation; NGO, non-governmental organisations; SRH, sexual and reproductive health; NCD, National Council for disability; CSOs, civil society organisations; FG, focus group.

## Discussion

 This paper emphasises the plurality of voices, the exploration of both problems and solutions, and the triangulation of methods. An important finding of this study is the convergence of views collected from policy actors at community and national levels, who identified multiple policy implementation challenges and barriers to SRH service use experienced by disabled users. From the study findings, we highlight learnings which emerged from our approach of using both an intersectional analysis and a participatory workshop to validate and enrich study findings. Study respondents referred to the principles of intersectionality related to knowledge, power, multilevel analysis, and the importance of context, equity, and reflexivity. Specifically, we address the following three points of discussion: (1) how diverse sources of knowledge and the reflexivity of policy actors can lead to new insight about their privileges and the discrimination and barriers faced by people with disabilities; (2) the importance of the post-conflict context in understanding policy implementation challenges and the experiences of barriers to access among people with disabilities; and (3) the capacity of policy actors to propose transformative solutions to redress health inequities faced by people with disabilities.

 First, through an intersectionality-informed analysis, we were able to analyse the different voices of different groups of policy actors. The study methodology capitalised on their distinctive social positions to shed light on their understanding of the relationships among legislation, policy and its implementation, and the use of SRH services by people with disabilities. Their views corroborated the perceptions of people with disabilities reported previously.^17^ People with disabilities experienced multiple physical, attitudinal, communication, and structural barriers. In particular, the sister study to this paper identified inequitable access to SRH services in health facilities and numerous intersectional discriminations related to gender, disability, and experience of violence.^17^ These barriers faced by people with disabilities have been discussed in the literature regarding Uganda,^15,16,35^ other sub-Saharan African countries,^36-39^ and globally.^40^ Furthermore, the interviewed policy actors were reflexive about their privilege, and the effects of oppression created by their inconsideration of the needs of people with disabilities. They recognised the effects that these internalised biases had on the experiences of access to and use of SRH services by users with disabilities.^41,42^ According to the IBPA principles, acknowledging the diverse sources of knowledge and highlighting the reflexivity of policy actors enable them to reflect upon the power and privilege they own.^42^ This realisation is a further step toward health equity and acts as a catalyst toward social justice.^42^

 Second, the post-conflict context in Northern Uganda was considered in our analysis. Our findings showed that time spent in the camps during and after the armed conflict and the post-conflict period has heavily affected Ugandans. The post-conflict continues to disadvantage people with disabilities in the Northern region, up to the current day. In an intersectionality approach, time and space (context) are key components in analysis.^42,43^ Literature has reported that the armed conflict in Uganda has caused limited access to and poor quality of maternal and reproductive health services,^14^ while sexual and gender-based violence aggravated the physical and psychological health of women.^12^ According to a systematic review on the long-term effects of armed conflicts, such as in Uganda, findings reported two types of effects, direct and indirect. Direct long-term effects included the experience of violence of all forms, disability, illnesses, injuries, and torture. The indirect long-term effects were characterised by limited access to healthcare and education as well as social marginalisation.^44^ Specifically, a study conducted among people with disabilities in the Gulu region reported the negative effect the conflict had on the psychological and emotional health of people with disabilities who shared their traumatic experiences and difficult coping strategies.^45^ These findings also reported difficulties in accessing healthcare services, including rehabilitation, such as assistive devices, and mental health services.^45^ Literature further mentioned that people with disabilities, especially women, faced discrimination and lacked access to health facilities upon return home, coupled with economic challenges.^46^

 Third, policy actors identified recommendations to the numerous barriers to SRH service utilisation experienced by people with disabilities, disability-focused policy implementation challenges, and multipronged recommendations addressed to policy actors at micro, meso, and macro levels. In an intersectionality-informed analysis, exploring alternatives and solutions is as important as identifying problems which need to be addressed.^20^ Reflecting upon and consciously proposing solutions is integral to a transformative process and contributes to eventually reaching equity and social justice. For example, the recommendation made by policy actors to allocate more budget on disability issues and to reinforce the position of the NCD found an echo in the revised 2019 Disability Act.^11^ Whereas the 2006 Disability Act did not include the scope of the NCD, the 2019 iteration of the Act specified its roles and funds, in addition to making the provision for representatives of the Council to work at the district level to enhance the presentation of people with disabilities in the community. Through the participatory workshop, health service providers and managers discovered the strengths of people with disabilities and that they could be experts in helping them devise health services to be more accessible and act as role models for others. While policy actors used to consider people with disabilities as weak and not capable, Intersectionality enabled them to acknowledge the multiple social categories a person/group may have, recognising that they may be simultaneously privileged in one context and be disadvantaged in another one.^42^

###  Limitations

 Given the richness of information elicited from different groups of policy actors, we were not able to report them all in a single manuscript. Comprehensive description and analyses from people with disabilities at individual level have been reported elsewhere,^17^ and the perceptions of policy actors at meso and macro levels are reported separately here. The contrasting of convergent or divergent views of policy actors will subsequently be discussed more in detail. We also did not include the views of policy actors located in other Northern districts which have been affected by the armed conflict. This inclusion may have expanded the depth of data collected and the richness of description to analyse. With more time and resources, this expansion would be possible. Given the convergence of problems and recommendations reported by study respondents, social desirability could have been a bias. However, respondents were clear about the observed multiple barriers faced by people with disabilities and the policy implementation challenges. They demonstrated the readiness to address these issues, collectively. Finally, to reduce the limitations of translation when it was used, we elaborated a glossary of research and SRH terms in English and Luo for consistency. Both research assistants were present during all interviews to support one another for translation when needed. At the end of each day of interviews, the research team met and debriefed about the interview process, including translation, for improvement purposes.

## Conclusion

 This study reveals the multilayered perceptions of policy actors at meso and macro levels of the relationships among pro-disability policy and legislation and the use of SRH services by people with disabilities in three post-conflict Northern districts of Uganda. The study findings intersect with and complement the perceptions and recommendations provided by people with disabilities at micro level. An intersectionality-informed analysis emphasised the importance of going beyond the identification of problems by concomitantly searching for solutions. With the recent adoption of the revised Disability Act in 2019, Uganda has renewed its commitment to remove barriers structurally and better protect the rights of people with disabilities. This creates a normative space for actions such as those recommended by the participants in our study. Concrete recommendations included empowering people with disabilities, families, and their organisations through awareness-creation and capacity-building, at micro level. At meso level, policy actors recommended training of health service providers on disability-sensitive services such as sign language, improving physical, attitudinal, and communication accessibility in health facilities, and collecting and analysing data on disability more systematically. At macro level, more accountability of policy-makers, active monitoring, and enforcing of policy implementation with disability budgeting were identified. The proposed solutions targeting three levels of policy actors, vertically, and various types of groups, horizontally, are within the reach and capacity of Government policy-makers, CSOs’ managers, health decision-makers, DPO leaders, and people with disabilities. As suggested by the UN report on the Sustainable Development Goals for people with disabilities,^5^ the recommendations can constitute the foundation for a hands-on road map to health equity by removing multiple barriers to access to and use of SRH services by people with disabilities, irrespective of their geographic location in Uganda.

## Acknowledgements

 We thank the study research assistants, Bryan Eryong and Emma Ajok, and all study participants and stakeholders. We are grateful to the St-Mary’s Hospital Lacor for its collaboration during fieldwork.

## Ethical issues

 This study received ethics approval from the *Centre de recherche du Centre hospitalier de l’Université de Montréal* (CR-CHUM) (17.127-CÉR, 1 August 2017); the Research Ethics Committee in Sciences and Health of the Université de Montréal (CERCES-20-074-D, 13 May 2020), following a change of research affiliation in Canada; the Lacor Hospital Institutional and Research Ethics Committee (LHIREC - 019/07/2017); and the Uganda National Council for Science and Technology (SS-4451, 14 November 2017).

## Competing interests

 Authors declare that they have no competing interests.

## Authors’ contributions

 MMS conceptualised the manuscript and collected and analysed the data. All authors contributed to, reviewed, read, and approved the final manuscript.

## Funding

 The authors thank the MoCHeLaSS (Mother Child health Lacor-South Sudan)/IDRC/IMCHA Project for its support. The MoCHeLaSS Project was carried out with the aid of a grant from the Innovating for Maternal and Child Health in Africa initiative- a partnership of Global Affairs Canada (GAC), the Canadian Institutes of Health Research (CIHR) and Canada’s International Development Research Centre (IDRC). MMS received a doctoral training scholarship from the Fonds de Recherche du Québec – Santé [0000256736] and a doctoral award from the International Development Research Centre: [Grant Number 108544-010]. The funding sources had no role in the study design, data collection, analysis, and interpretation, or writing and preparation of the manuscript, or decision to publish.
